# mlDEEPre: Multi-Functional Enzyme Function Prediction With Hierarchical Multi-Label Deep Learning

**DOI:** 10.3389/fgene.2018.00714

**Published:** 2019-01-22

**Authors:** Zhenzhen Zou, Shuye Tian, Xin Gao, Yu Li

**Affiliations:** ^1^Computer, Electrical and Mathematical Sciences and Engineering (CEMSE) Division, Computational Bioscience Research Center (CBRC), King Abdullah University of Science and Technology (KAUST), Thuwal, Saudi Arabia; ^2^Department of Biology, Southern University of Science and Technology (SUSTC), Shenzhen, China

**Keywords:** multi-functional enzyme, function prediction, EC number, deep learning, hierarchical classification, multi-label learning

## Abstract

As a great challenge in bioinformatics, enzyme function prediction is a significant step toward designing novel enzymes and diagnosing enzyme-related diseases. Existing studies mainly focus on the mono-functional enzyme function prediction. However, the number of multi-functional enzymes is growing rapidly, which requires novel computational methods to be developed. In this paper, following our previous work, DEEPre, which uses deep learning to annotate mono-functional enzyme's function, we propose a novel method, mlDEEPre, which is designed specifically for predicting the functionalities of multi-functional enzymes. By adopting a novel loss function, associated with the relationship between different labels, and a self-adapted label assigning threshold, mlDEEPre can accurately and efficiently perform multi-functional enzyme prediction. Extensive experiments also show that mlDEEPre can outperform the other methods in predicting whether an enzyme is a mono-functional or a multi-functional enzyme (mono-functional vs. multi-functional), as well as the main class prediction across different criteria. Furthermore, due to the flexibility of mlDEEPre and DEEPre, mlDEEPre can be incorporated into DEEPre seamlessly, which enables the updated DEEPre to handle both mono-functional and multi-functional predictions without human intervention.

## 1. Introduction

Enzymes, which catalyze reactions *in vivo*, play a vital role in metabolism in every species. Predicting enzyme function is an important bioinformatics task, for helping researchers design more efficient novel enzymes and assisting people in diagnosing enzyme-related diseases (Hoffmann et al., 2007). To predict enzyme function, a clear and standard enzyme function ontology should be defined. Currently, the most popular way of standardizing enzyme function is to use the EC number system (Cornish-Bowden, 2014). An enzyme commission (EC) number is composed of 4 digits, i.e., EC 3.1.21.4, with the first digit denoting the main class of the enzyme; and the second digit indicating the subclass of the enzyme, etc. Each further digit defines the function of an enzyme more specifically, combining with the previous digits. As shown in Figure 1 in Shen and Chou (2007), the label space of the EC system has a tree structure. As an important bioinformatics task, a number of methods have been proposed to deal with the problem, based on structure similarity (Dobson and Doig, 2005; Roy et al., 2012; Yang et al., 2015), sequence similarity (Tian et al., 2004; Quester and Schomburg, 2011), or machine learning techniques (des Jardins et al., 1997; Cai et al., 2003; Shen and Chou, 2007; Zhou et al., 2007; Li et al., 2018d). Despite the success of those methods in predicting mono-functional enzyme function with very high accuracy, seldom have people worked on the prediction of multi-functional enzyme function, which actually constitutes a relatively large part of all the enzymes. Until now, to our knowledge, only five methods (De Ferrari et al., 2012; Zou et al., 2013; Che et al., 2016; Zou and Xiao, 2016; Amidi et al., 2017) are able to address that specific type of enzymes. Among them, De Ferrari et al. (2012) use InterPro signatures as the features and multi-label k-nearest neighbor (KNN) as the algorithm. Zou et al. (2013) took advantage of a number of manually designed features, such as a 20-D feature vector extracted from the position-specific scoring matrix (PSSM) and a 188-D feature vector based on the composition and physical-chemical properties of the protein, and the conventional multi-label machine learning algorithm. Che et al. (2016) also utilized features extracted from PSSM, combined with the multi-label KNN algorithm. Zou and Xiao (2016) deployed three variants of the famous feature, Pseudo Amino Acid Composition (PseAAC), and the multi-label KNN algorithm. Amidi et al. (2017) used the predicted structure information, combined with the sequence information, as the feature, and multi-label KNN and multi-label support vector machine (SVM) as the classifier. Despite their satisfactory performance on a specific dataset, we can find that all of those algorithms can be improved in two ways. Firstly, all the methods utilize very specific expert-designed features. It is both time and knowledge consuming to do so, and the extracted features can be local minima for this particular problem (Dai et al., 2017; Li et al., 2018d). The features automatically designed and extracted from the raw representation of a protein by an algorithm are more desirable than the human designed features. Secondly, almost all the aforementioned methods rely on KNN. KNN is a similarity based algorithm. Unlike the probability based algorithms, KNN is unable to annotate novel enzymes which do not have homologs with high sequence similarities in the current databases. Both the feature design process and the classification process have great potential to be improved.

**Figure 1:**
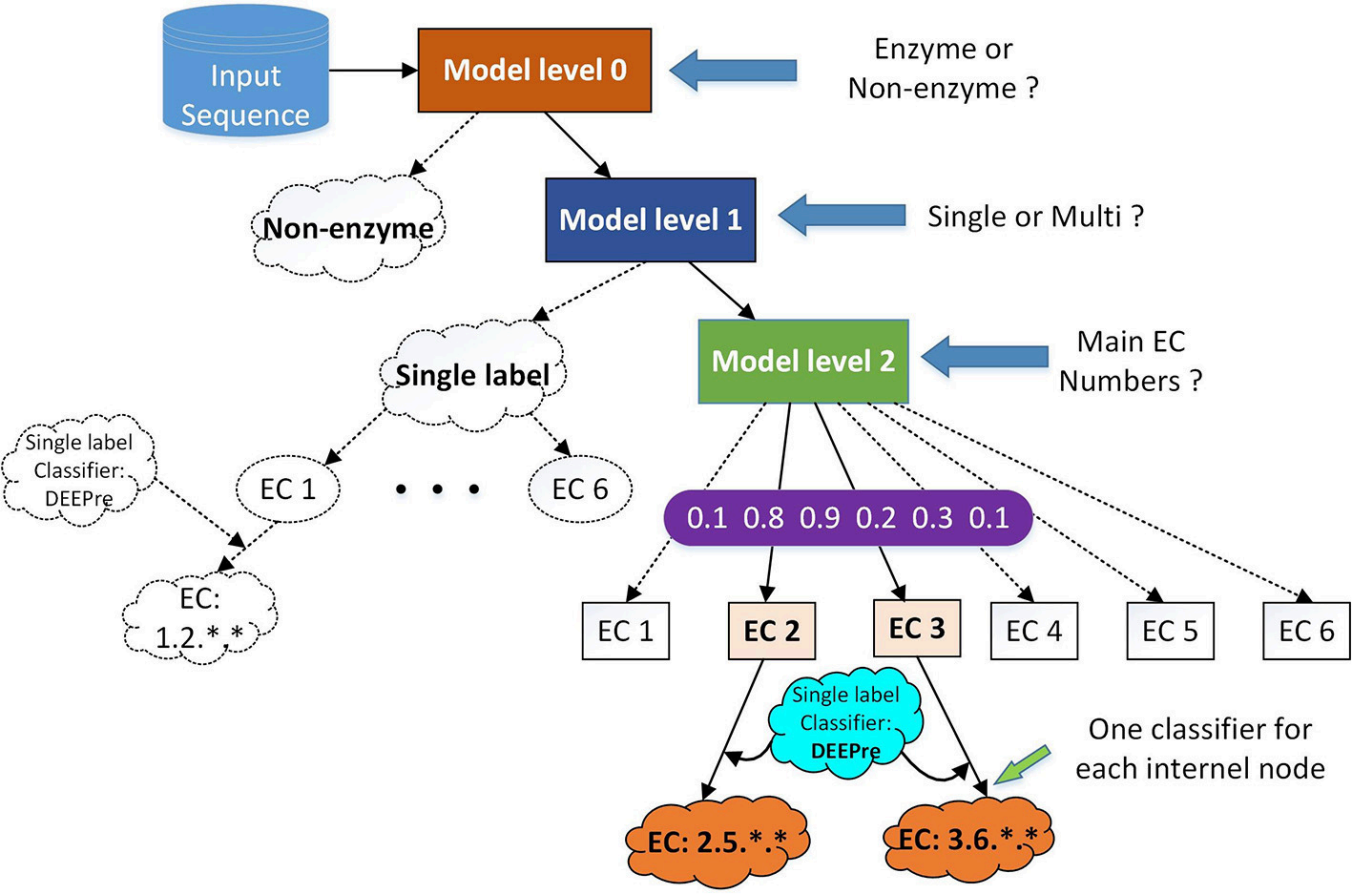
The hierarchical classification strategy combining DEEPre and mlDEEPre. When we input a protein sequence to the system, we first use DEEPre level 0 to predict whether the input sequence is an enzyme or not. If it is an enzyme, we use mlDEEPre level 1 to predict whether the enzyme is a mono-functional enzyme or a multi-functional enzyme. If it is a mono-functional enzyme, DEEPre will take over. If not, we use mlDEEPre to predict that multi-functional enzyme's main classes. Inputting the main classes and the sequence to DEEPre, we can obtain the full annotation for each function of the enzyme.

On the other hand, deep learning (LeCun et al., 2015), an end-to-end learning algorithm which wraps the representation learning and classifier learning into one model, has shown great potential in the bioinformatics field (Dai et al., 2017; Li et al., 2018a,d,e; Umarov et al., 2018; Xia et al., 2018), especially in the enzyme function prediction direction (Li et al., 2018d), in which the newly proposed deep learning based method, DEEPre (Li et al., 2018d), has improved the state-of-the-art performance significantly. In general, Li et al. (2018d) built one deep learning model for each of the internal nodes in the tree structure of the EC number system. In particular, for level 0, that is, predicting whether the input protein sequence is an enzyme or not, there is one model; for level 1, whose task is to predict the main class of an enzyme from the six classes, there is one model; for level 2, which predicts the subclass of an enzyme, there are six models since there are six main classes and we need to build a model for each of those different main classes. In terms of the deep learning model architecture, Li et al. (2018d) proposed a novel architecture which can extract convolutional information and sequential information from three raw representations (PSSM, sequence encoding and functional domain encoding) and combine them together automatically for the downstream classification. They only fed the very raw encoding of the protein sequence to the deep learning model and the model is responsible for both feature extraction and classification. In this way, the algorithm is likely to find a better hidden feature representation implicitly, which benefits the classification results.

However, despite the success of DEEPre, the original version of DEEPre was designed specifically for mono-functional enzyme function prediction and not capable of handling multi-functional enzymes. Following the success of DEEPre and its research direction, we propose a novel hierarchical multi-label deep learning method, mlDEEPre, for predicting the multi-functional enzyme functions. In particular, mlDEEPre first predicts whether an enzyme is a mono-functional enzyme or a multi-functional enzyme as a binary classification problem. If the enzyme is a multi-functional enzyme, it will take the input enzyme sequence and predict its main classes as a multi-label prediction problem. To equip the deep learning model with multi-label prediction ability, we adopt the idea of backpropagation for multilabel learning (BP-MLL) (Zhang and Zhou, 2006) into the original DEEPre architecture. Meanwhile, since the entire DEEPre package can also take the main class of the sequence as input and start the prediction from the second level, after obtaining the main classes of the multi-functional enzymes, we can feed the mlDEEPre result to DEEPre, predicting all the four digits for each function of an enzyme. In this work, we make the following contributions:

- We extend the capability of DEEPre from mono-functional enzyme function prediction to multi-functional enzyme function prediction.
- We propose a novel multi-label deep learning framework based on BP-MLL which can be useful for other multi-label prediction problems in bioinformatics.

## 2. Materials and Methods

In this section, we first introduce the dataset used to evaluate the proposed method (section 2.1) and the needed raw encodings we feed to the deep learning model (section 2.2). Then, we provide a big picture of the mlDEEPre method in section 2.3. After that, we introduce the deep learning architecture used in our method (section 2.4). Following the model introduction, we describe how we equip the model with the ability to perform multi-label prediction (section 2.5 and section 2.6). Finally, we wrap up the mlDEEPre method and combine it with the original version of DEEPre (section 2.7).

### 2.1. Dataset

For the mono-functional enzyme data, we use the dataset from Li et al. (2018d). As for the multi-functional enzyme data, we use the dataset from Che et al. (2016). Li et al. (2018d) constructed a dataset containing 22,168 sequences from UniProt which have mono-function with 40% sequence similarity filtered by CD-hit. Che et al. (2016) provided us with 4,076 multi-functional enzymes. More detailed descriptions of how to construct the datasets can be referred to Li et al. (2018d) and Che et al. (2016). Here, we provide the statistics of the mono-functional and the multi-functional enzyme datasets in Tables 1–3.

**Table 1:** Dataset I: 22,168 single-labeled enzymes.

**Class**	**EC 1**	**EC 2**	**EC 3**	**EC 4**	**EC 5**	**EC 6**
Name	Oxidoreductase	Transferase	Hydrolase	Lyase	Isomerase	Ligase
Number	3343	8517	5917	1532	1193	1666

**Table 2:** Dataset II: 4,076 multi-labeled enzymes.

**Number of classes**	**2**													**3**			**4**
EC numbers	1	1	1	1	2	2	2	2	3	3	3	4	4	1	1	1	1
														2	3	4	2
	2	3	4	5	3	4	5	6	4	5	6	5	6	4	6	5	3
																	4
Number	147	841	63	37	1148	235	38	131	622	22	4	308	34	215	10	211	10

*This table shows the number of multi-functional enzymes in the dataset with different EC main class combinations*.

**Table 3:** Dataset II: 1,085 multi-labeled enzymes with 65% sequence similarity cut-off.

**Multifunctional enzymes**	**EC 1**	**EC 2**	**EC 3**	**EC 4**	**EC 5**	**EC 6**	**Total**
Name	Oxidoreductase	Transferase	Hydrolase	Lyase	Isomerase	Ligase	
Before redundancy	1534	1924	2657	1698	616	179	4076
After CD-HIT	386	503	689	473	137	52	1085

### 2.2. Protein Raw Encoding

Similar to DEEPre (Li et al., 2018d), we use the following three raw protein encodings to represent a protein sequence, which will be fed to the deep learning model as inputs.

#### 2.2.1. PSSM

For each protein sequence, we run PSI-BLAST (Altschul et al., 1997) from BLAST+ (Camacho et al., 2009) against SWISS-PROT (Bairoch and Apweiler, 2000), with three iterations and the e-value as 0.002, to find the sequence homologies. Then we align those sequences, and for each position in the query protein, calculate a vector which indicates the appearance frequency of each amino acid in the alignment. The evolutionary information of the protein sequence is encoded by an L by 20 matrix.

#### 2.2.2. Sequence One-Hot Encoding

To represent the original protein sequence information, we use one-hot encoding. For each type of amino acids, we use a vector composed with nineteen 0s and one 1 to represent it. For example, ‘A’ is represented as (1, 0_1_, …, 0_19_) and ‘C’ is represented as (0_1_, 1, …, 0_19_). In this way, each position of the protein sequence is encoded into a vector. Putting those vectors together, we have an L by 20 matrix to represent the original raw sequence.

#### 2.2.3. Functional Domain Encoding

This representation encodes the functional domain within a protein sequence. We use HMMER (Eddy, 2011) to search a query protein against Pfam (Finn et al., 2016), which is a functional domain database. If one functional domain is hit, we use 1 to encode it; otherwise, we use 0 to encode it. Consequently, we have a vector composed of 0s and 1s to show the functional domain information of a protein.

### 2.3. mlDEEPre

The primary task of mlDEEPre is to predict the main classes of multi-functional enzymes. However, we start from predicting whether a query enzyme is a multi-functional enzyme or not. As shown in Figure 1, mlDEEPre has two levels. Given an enzyme sequence, the first level predicts whether the enzyme is a mono-functional enzyme or a multi-functional one. If the sequence is a multi-functional enzyme, the second level of mlDEEPre will predict the main classes of the enzyme's multi-functions. The model architecture of the two levels is discussed in section 2.4 and the specific design for multi-label prediction is discussed in sections 2.5 and 2.6. mlDEEPre has very close relationship with the original version of DEEPre, which is discussed in details in section 2.7.

### 2.4. Model Architecture

**Figure 2:**
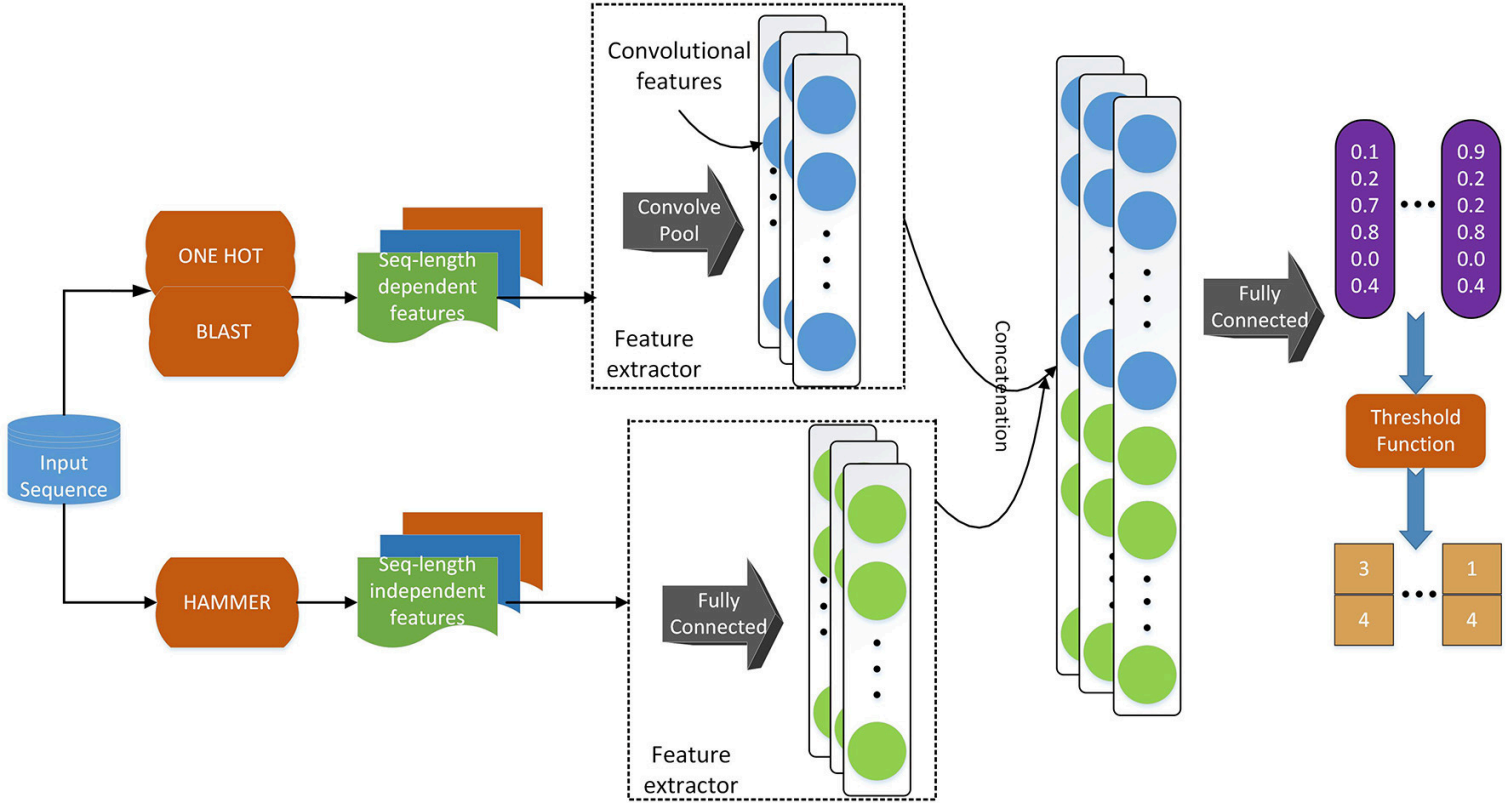
The deep learning model architecture. We use a convolutional neural network component to deal with sequence-length dependent features, such as PSSM and sequence one-hot encoding, and fully connected neural network component to handle functional domain encoding. After those components, we concatenate their outputs into one vector, which is fed to a fully connected classifier. We apply a threshold function to the output of the model to obtain the labels of the input sequence.

Regarding the deep learning model, we use a similar model architecture as in Li et al. (2018d). As shown in Figure 2, we adopt convolutional layers for the sequence-length dependent features, i.e., PSSM and sequence one-hot encoding, to extract useful information from those encodings. Since functional domain encoding has already been a high level feature with a fixed length, we use fully-connected layers to reduce the dimensionality and further extract information. After those separated layers for each feature, we concatenate their outputs and feed the concatenated vector to a fully connected layer, which can be considered as the classifier. Training the model in an end-to-end manner, we are able to optimize the feature extractors (the layers before concatenation) and the classifiers (the final fully connected layers) at the same time, resulting in a better hidden feature representation and thus a classification model with better performance.

### 2.5. Multi-Label: Loss Function

Deep learning methods are often suitable for multi-label classification. As shown in Figure 2, the model's last layer has multiple nodes, whose outputs correspond to the predicted probability of each label. If we use the model to perform single label classification, we will find the label with the highest probability score and assign the query with that label. When we use the model to perform multi-label prediction, we can still use the predicted probabilities. However, we need to change the way of assigning labels. Instead of assigning the label with the highest probability, we may want to assign the labels whose probability score is higher than a certain threshold so that multiple labels can be predicted. On the other hand, when we train the model, we also need to consider the multi-label information in the training data. One of the most straightforward way of incorporating such information when training the model is by changing the loss function to let the model know that we are performing multi-label prediction using a multi-label dataset. In terms of such threshold and loss function, we adopt the idea from Zhang and Zhou (2006), i.e., BP-MLL. We introduce the loss function in this section in details and discuss the threshold in the next section.

Formally, denote the *i*^*th*^ enzyme instance as *x*_*i*_, and its corresponding label vector as *D*_*i*_. Each element of *D*_*i*_ is a binary value, which indicates whether that enzyme instance belongs to a certain class. We use $d_{i}^{j}$ to denote that element, where *j*∈[1, 6] for our problem. If $d_{i}^{j}$ is 1, the enzyme *x*_*i*_ belongs to the class *j*, 0 otherwise. As for a classification problem, the most intuitive way to define the global error of the network is to measure the distance between the predicted labels and the real labels of the training set:

(1) $$\begin{array}{r} E=\overset{m}{\underset{i=1}{\sum }}E_{i}, \end{array}$$

where *E*_*i*_ represents the network error on the instance *x*_*i*_ and *m* is the size of the training data. For a multi-label classification problem, we can define *E*_*i*_ as below:

(2) $$E_{i}=\sum_{j\;=\;1}^{Q}{\left(l_{i}^{j}-d_{i}^{j}\right)}^{2},$$

where $l_{i}^{j}$ and $d_{i}^{j}$ are the actual output and the true label of the network on *x*_*i*_ on the class *j*, respectively; Q is the total number of classes, which is 6 in our problem. Using Equation 2, we are able to incorporate the multi-label information into the model to a certain degree since all the label information is considered in that loss function. However, the loss function in Equation 2 assumes that each class label is independent, which ignores any relationship between different class labels. In reality, one of the most straightforward relationships between labels is that labels in $L_{i}^{true}$ should have higher ranks than those not in $L_{i}^{true}$, where $L_{i}^{true}$ is set of labels that the instance *x*_*i*_ has. Accordingly, we can use the following function as the loss which considers the rank relationship between labels:

(3) $$\begin{array}{r} E=\overset{m}{\underset{i=1}{\sum }}E_{i}=\overset{m}{\underset{i=1}{\sum }}\frac{1}{\left|L_{i}^{true}||\bar{L_{i}^{true}}\right|}\underset{\left(k,q\right)\in L_{i}^{true}\times \bar{L_{i}^{true}}}{\sum }e^{\left(-\left(l_{i}^{k}-l_{i}^{q}\right)\right)}, \end{array}$$

where $\bar{L_{i}^{true}}$ is the complementary set of $L_{i}^{true}$, that is, the label set which the instance *x*_*i*_ does not have, and |•| is the cardinality measure of a set. From the equation, we can find that $\left(l_{i}^{k}-l_{i}^{q}\right)$ measures the difference between the outputs of the network on the labels belonging to the training instance and the ones not belonging to it, which is further fed to the exponential function. When $l_{i}^{q}$ happens to be much larger than $l_{i}^{k}$, which causes large discrepancy, the exponential function can penalize the error severely. By minimizing Equation 3, we can make the model output much higher values for the true labels while very small values for the labels that the training data do not have. Thus, labels in $L_{i}^{true}$ have higher ranks than those not in $L_{i}^{true}$, which is in agreement with our goal.

### 2.6. Multi-Label: Threshold

As discussed in the previous section, when we use the model, to determine and assign the labels, there should be a threshold *t*(*x*), which is applied to the output of the deep learning model, so that we predict the labels as $L_{i}^{pred}=\left\{j|l_{i}^{j}>t\left(x\right),j\in \left[1,6\right]\right\}$. A straightforward and natural solution of the threshold function is to set *t*(*x*) as a constant. However, that constant threshold does not consider the difference between different data points. To solve the problem, Elisseeff and Weston (2002) proposed an excellent idea to incorporate the information of each single data point into the threshold, which replaces the constant with a linear function $t\left(x_{i}\right)={\text{w}}^{⊺}\cdot l\left(x_{i}\right)+b$, where *l*(*x*_*i*_) is the output of the network on the instance *x*_*i*_. In this way, each data point can have its own threshold, which is more flexible than a constant. To obtain the threshold function, we need to solve the following problem:

(4) $$\begin{array}{r} t\left(x_{i}\right)=argmin_{t}\left(\left|\left\{k|k\in L_{i}^{true},l_{i}^{k}⩽t\right\}|+|\left\{q|q\in \bar{L_{i}^{true}},l_{i}^{q}⩾t\right\}\right|\right). \end{array}$$

If the solution of Equation 4 is not unique and the solution composes a segment, the middle value of the value range is chosen as the threshold. For example, assume the real label and predicted label set of *x*_*i*_ are {1,1,0,0,0,0} and {0.9, 0.8, 0.3, 0.1, 0.1, 0.1}, when 0.3 < *t* < 0.8, $\left|\left\{k|k\in L_{i}^{true},l_{i}^{k}\leq t\right\}|+|\left\{q|q\in \bar{L_{i}^{true}},l_{i}^{q}\geq t\right\}\right|$ always takes the minimum value as 0. Consequently, we choose the middle value of (0.3, 0.8), which is 0.55, as the threshold. In BP-MLL, the solution of the threshold equation can be obtained through the linear least square method.

To sum up, after we have a well-trained model and the threshold function parameters, and when we need to use the model to perform prediction, firstly, we feed the test instance to the trained network and get the outputs *l*(**x**). Secondly, we calculate the threshold using *t*(**x**) = **w**^⊺^ · *l*(**x**) + *b* and apply the threshold to the output of the model, obtaining the predicted labels for the enzyme instance *x*.

### 2.7. DEEPre and mlDEEPre

Although DEEPre is designed for mono-functional enzyme function prediction, it is very flexible, being able to predict the detailed function of an enzyme from the first level or the second level. For example, if we have already known that an enzyme has the follow incomplete EC number: 1.-.-.-, we can run DEEPre from the second level to fulfill the missing digits. Taking into consideration the enzyme's feature representation and the fact that the query sequence is an Oxidoreductase, we run the model trained specifically for the enzyme with the first EC digit as 1. With such flexibility, we can easily combine mlDEEPre and DEEPre to predict the detailed functionality of multi-functional enzymes. Using mlDEEPre, we can predict the main classes of those multi-functional enzymes, such as 2.-.-.- and 3.-.-.-. Feeding the sequence and the main classes annotation to DEEPre, we are able to fill in the missing digits for each incomplete annotation of a multi-functional enzyme. The idea of combining DEEPre and mlDEEPre is illustrated in Figure 1. Starting from a protein sequence, we first use level 0 of DEEPre to predict whether the protein is an enzyme or not. If yes, we use mlDEEPre first level to predict whether the enzyme is a mono-functional enzyme or multi-functional enzyme. If that is a mono-functional enzyme, we will further run DEEPre to get full annotation of that enzyme. If not, we will run the second level of mlDEEPre to predict the main classes of the enzyme. For each function, we run DEEPre to obtain the full annotation. Considering that most multi-functional enzymes have multiple EC number annotations for its different functions diverging in the first digit, our method is efficient and reliable under most circumstances.

## 3. Results

In this section, we first briefly introduce the methods with which we are going to compare mlDEEPre (section 3.1). And then, we define the evaluation criteria for the comparison in details in section 3.2. After that, we show the performance of our method in predicting whether an enzyme is a mono-functional enzyme or a multi-functional enzyme in section 3.3. Furthermore, section 3.4 gives the main classes prediction results of multi-functional enzymes. Finally, we show our method's performance on fatty acid synthase (FAS) function prediction in section 3.5.

### 3.1. Compared Methods

For mono-function prediction, we compared our method with Pse-ACC (Chou and Ho, 2006), ACC (Che et al., 2016), EnzML (De Ferrari et al., 2012), and SVM. Pse-ACC (Chou and Ho, 2006) is a widely used tool, which predicts the enzymatic attribute of proteins by considering the functional domain composition of a given enzyme sequence. In ACC (Che et al., 2016), the authors utilized autocross-covariance (ACC) feature representation which consists of two feature models, autocovariance (AC) and cross-covariance (CC) (Dong et al., 2009). Another compared method here is EnzML, which can efficiently utilize the InterPro signatures. All the above three methods used K-nearest neighbors (KNN) based classification algorithm as the base classifier. We also compared our method with a baseline method, which used SVM as the algorithm and ACC as the features.

For multi-function prediction, as discussed before, until now, there are only a few works focused on this problem, and all of them are based on KNN. The key idea of KNN is that similar instances should share the same labels and we can assign the labels to a query sequence with the most frequent ones from its K-most similar instances, the idea of which is shown in Figure S1. We compared our method with ML-KNN (Zhang and Zhou, 2007), BR-KNN (Spyromitros et al., 2008), IBLR-ML (Cheng and Hüllermeier, 2009), GM (Zou and Xiao, 2016), and SVM-NN (Amidi et al., 2017). In ML-KNN (Zhang and Zhou, 2007), for each unseen instance, its K-nearest neighbors in the training set are firstly identified. And then, maximum a posteriori estimation (MAP) principle is applied to determine the label set of the unseen instance based on the statistical information of its neighbor samples. As for Binary Relevance KNN (BR-KNN) (Spyromitros et al., 2008), it learns *M* binary classifiers, one for each class. In terms of instance-based learning and logistic regression (IBLR) (Cheng and Hüllermeier, 2009), it combines instance-based learning and logistic regression with ML-KNN. For the above three methods, the ACC (Dong et al., 2009) is used as the feature representation. Furthermore, Zou and Xiao (2016) utilized ML-KNN and a different feature extraction model, Grey Model (GM) (Lin et al., 2011), to perform the task. The last method is SVM-NN (Amidi et al., 2017). In this method, the authors combined structural and amino acid sequence information together, investigating two fusion approaches both in the feature level and the algorithm level (SVM and KNN), resulting in a method for general enzymatic function prediction.

### 3.2. Evaluation Criteria

#### 3.2.1. Single-Label Measurement

Given multi-label and mono-label test datasets $S=\left\{\left(x_{i},L_{i}^{true}\right)\right\}$, the binary classifier performance is evaluated by the four criteria: accuracy, precision, recall, and F1-score, which are defined below:

(5) $$\begin{array}{r} Accuracy=B\left(TP_{j},FP_{j},TN_{j},FN_{j}\right)=\frac{TP_{j}+TN_{j}}{TP_{j}+FP_{j}+TN_{j}+FN_{j}}, \end{array}$$

(6) $$\begin{array}{r} Precision=B\left(TP_{j},FP_{j},TN_{j},FN_{j}\right)=\frac{TP_{j}}{TP_{j}+FP_{j}}, \end{array}$$

(7) $$\begin{array}{r} Recall=B\left(TP_{j},FP_{j},TN_{j},FN_{j}\right)=\frac{TP_{j}}{TP_{j}+FN_{j}}, \end{array}$$

(8) $$\begin{array}{r} F1-score=\frac{2\;*\;Precision\;*\;Recall}{Precision+Recall}, \end{array}$$

in which *B*(*TP*_*j*_, *FP*_*j*_, *TN*_*j*_, *FN*_*j*_) represents the binary classification indicator; *TP*_*j*_ indicates the number of true positive instance; *TN*_*j*_ is the number of true negative instance; *FP*_*j*_ stands for the number of false positive instances; and *FN*_*j*_ represents the number of false negative instances.

#### 3.2.2. Multi-Label Measurement

Regarding the multi-label classification evaluation, the measurement criteria cannot be exactly the same as those in single-label classification. The assessment method is much more complicated in multi-label learning. The previous works (Chou and Ho, 2006; Zhang and Zhou, 2007) have defined various metrics, including example-based and label-based metrics. For example-based methods, the classification results for each instance are calculated first. After that, the average value for the entire dataset is obtained. For label-based metrics, the binary classification results for each class are calculated first, and then the average value for all classes is given. Here, we adopt both example-based and label-based methods. The metrics that have been utilized to assess the performance of mlDEEPre are described below:

##### 3.2.2.1. Hamming-loss

Hamming-loss evaluates the frequency of incorrect prediction of an instance-label pair. This index is averaged over all classes and the entire dataset. The smaller hamming loss is, the better the performance of the classifier is. It is defined as follows:

(9) $$\begin{array}{r} Hamming-loss=\frac{1}{N}\overset{N}{\underset{i=1}{\sum }}\frac{1}{6}|L_{i}^{pred}\Delta L_{i}^{true}{|}_{1}, \end{array}$$

where Δ is symmetric difference between two sets, |•|_1_ represents *l*_1_-norm, and N is the number of example enzymes.

##### 3.2.2.2. Subset accuracy

Subset accuracy is the strictest evaluation in multi-label classification. For each sample, the entire set of labels must be correctly predicted, otherwise the subset accuracy for that instance is equal to 0. In literature, it is also known as zero-one-loss:

(10) $$\begin{array}{r} Subset\;accuracy=\frac{1}{N}\overset{N}{\underset{i=1}{\sum }}\delta \left(L_{i}^{pred},L_{i}^{true}\right), \end{array}$$

where δ is the Kronecker delta:

(11) $$\{\begin{array}{cc} \delta (L_{i}^{pred},L_{i}^{true})=1, & \begin{array}{l} \;if\;and\;only\;if\;all\;the\;labels\;in\;L_{i}^{pred}\;\\ \;are\;equal\;to\;those\;in\;L_{i}^{true}, \end{array}\\ \delta (L_{i}^{pred},L_{i}^{true})=0, & otherwise. \end{array}$$

Opposite to hamming-loss, and just as the following Macro and Micro methods, the higher the subset accuracy is, the better the performance is.

##### 3.2.2.3. Macro-precision, Macro-recall, Macro-F1-score

Macro-precision, Macro-recall, and Macro-F1-score, which have been used in multi-label classification, calculate precision, recall, and F1-score separately for each class. The Macro-average method is straightforward: just take the average of the precision and recall of the system on different classes. When we want to evaluate system performance on different datasets, macro-averaged metrics are the best choice:

(12) $$\begin{array}{r} Macro-precision=\frac{1}{6}\overset{6}{\underset{j=1}{\sum }}\frac{TP_{j}}{TP_{j}+FP_{j}}, \end{array}$$

(13) $$\begin{array}{r} Macro-recall=\frac{1}{6}\overset{6}{\underset{j=1}{\sum }}\frac{TP_{j}}{TP_{j}+FN_{j}}, \end{array}$$

(14) $$\begin{array}{r} Macro-F1-score=\frac{2}{6}\overset{6}{\underset{j=1}{\sum }}\frac{Macro-precision_{j}\times Macro-recall_{j}}{Macro-precision_{j}+Macro-recall_{j}}. \end{array}$$

##### 3.2.2.4. Micro-precision, Micro-recall, Micro-F1-score

Regarding Micro-precision, Micro-recall, and Micro-F1-score, in Micro-average methods, we sum up the individual TP, FP, TN, and FN of the system for different sets and then apply them to get the statistics. The Micro-metrics pay more attention to whether the enzymes are correctly classified, regardless their original distribution. Thus, in case of the dataset size being variable, Micro-averaged indexes are the better choice:

(15) $$\begin{array}{r} Micro-precision=\frac{\sum_{j=1}^{6}TP_{j}}{\sum_{j=1}^{6}TP_{j}+\sum_{j=1}^{6}FP_{j}}, \end{array}$$

(16) $$\begin{array}{r} Micro-recall=\frac{\sum_{j=1}^{6}TP_{j}}{\sum_{j=1}^{6}TP_{j}+\sum_{j=1}^{6}FN_{j}}, \end{array}$$

(17) $$\begin{array}{r} Micro-F1-score=2\cdot \frac{Micro-precision\times Micro-recall}{Micro-precision+Micro-recall}. \end{array}$$

### 3.3. Mono-Functional vs. Multi-Functional Prediction

**Figure 3:**
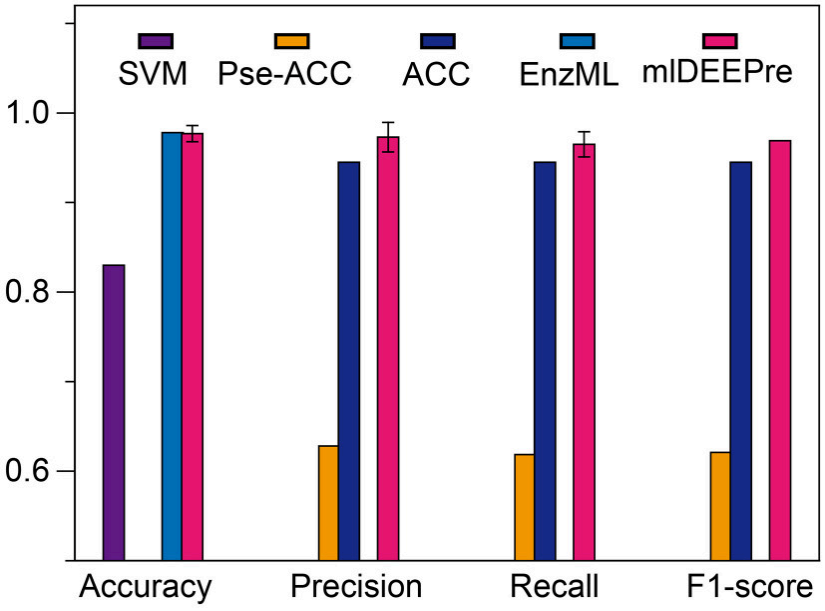
The mono-functional enzyme VS multi-functional enzyme classification testing performance of different models. Performance lower than 0.6 are not shown in the figure.

In this section, we describe the performance of the proposed method in predicting whether an enzyme is a mono-functional or multi-function enzyme. The training and testing datasets used in this work are shown in Tables 1, 3. It is worthy pointing out that the data are imbalanced, with 22,168 mono-functional enzymes and 1085 multi-functional enzymes. In this work, we employed penalized models to overcome the imbalance, forcing the model to pay more attention to the multi-functional class. We ran the model 30 times on a GPU node with 32 CPU cores and one GTX 1080 Ti card, each time with 70% of all the data as training data and 30% as testing data. The average training time is 11 h for 40 epochs and the average testing time for one batch is < 1 min. We show the comparison results, both the average and the standard deviation, in Figure 3. As suggested by Figure 3, our method can outperform all the other methods consistently across different criteria. Besides, our method is very stable, with the standard deviation of accuracy being as low as 0.09.

### 3.4. Multi-Functional Enzyme Main Classes Prediction

**Figure 4:**
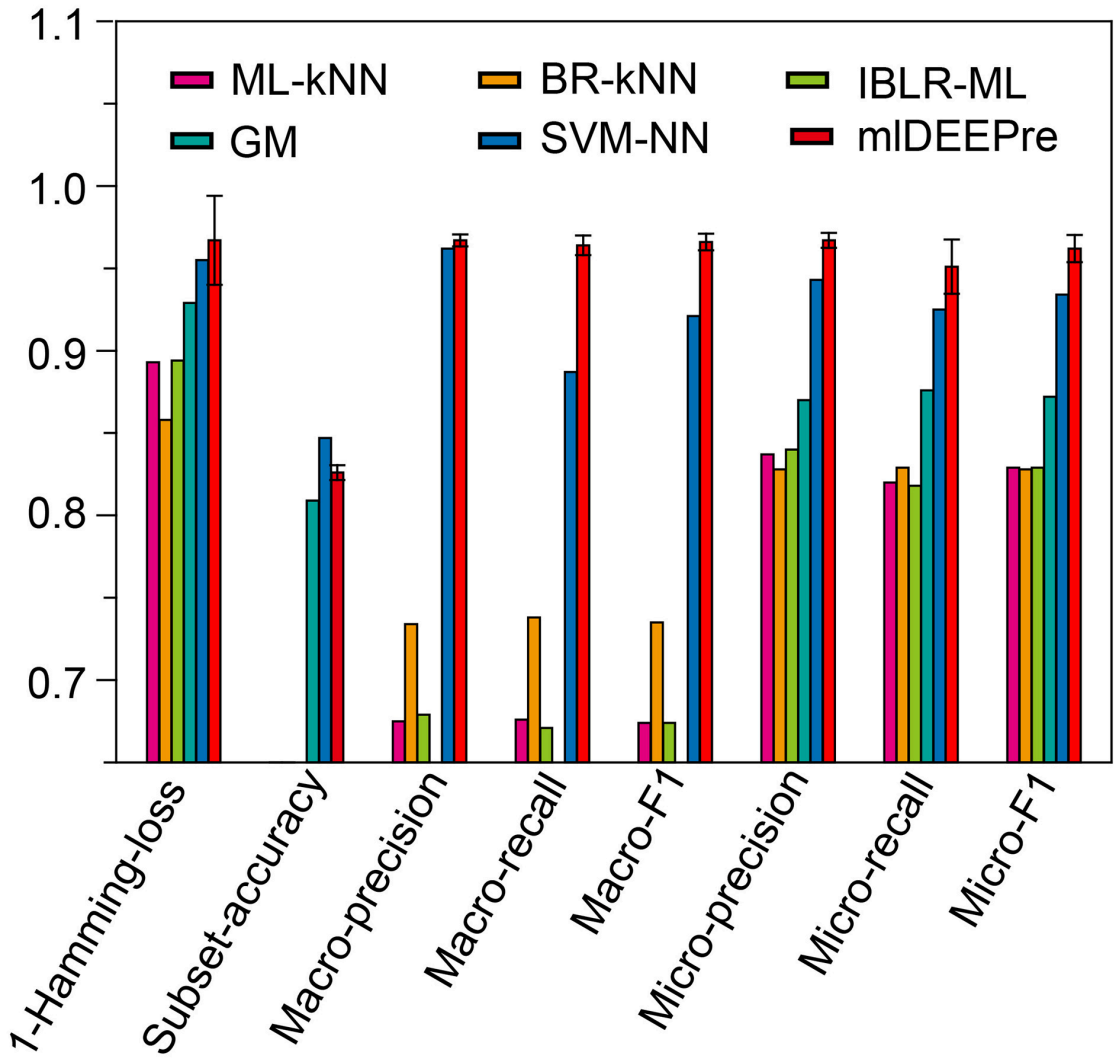
The multi-functional classification testing performance of different models. Performance lower than 0.65 are not shown in the figure.

Similar to the experiments in section 3.3, we also ran the model 30 times on a GPU node with 32 CPU cores and one GTX 1080 Ti card, each time with 70% of all the data as training data and 30% as testing data. The average training time is about 14 h for 40 epochs and the average testing time for one batch is around one minute. Using the criteria for multi-label learning that have been discussed in section 3.2, we evaluated mlDEEPre and compared it with other models introduced in section 3.1, obtaining the performance results shown in Table 4 and Figure 4. According to Hamming-loss, the proposed multi-label model, mlDEEPre, predicts 97.6% of all the actual main classes in the test dataset correctly, with the corresponding standard deviation being 2.7%, which outperforms all the other methods. Furthermore, we also compared our method with the other methods using other criteria. Although, because SVM-NN is good at predicting those rare class labels caused by imbalanced training samples (only 52 sequences belonging to class 6), the performance of SVM-NN (84.7%) is slightly better than that of mlDEEPre (82.6%) and GA (80.8 %) in subset accuracy, mlDEEPre performs better than all the other methods in term of all the other criteria, including Macro-precision, Macro-recall, Macro-F1, Micro-precision, Micro-recall, and Micro-F1.

**Table 4:** The multi-functional classification performance of mlDEEPre on dataset II shown in Table 3.

**Hamming-loss**	**Subset accuracy**	**Macro-precision**	**Macro-recall**
3.3 ± 0.4%	82.6 ± 2.7%	96.7 ± 0.3%	96.4 ± 0.6%
**Macro-F1**	**Micro-precision**	**Micro-recall**	**Micro-F1**
96.5 ± 0.5%	96.7 ± 0.4%	95.1 ± 1.6%	96.2 ± 0.8%

### 3.5. Case Study: FAS

Fatty acid synthase (FAS) is a homodimeric multi-functional enzyme that performs the anabolic conversion of dietary carbohydrate or proteins to fatty acids (Chakravarty et al., 2004). Many human cancers can cause high level expression of FAS. Meanwhile, the regulation of human FAS in a variety of cancers makes FAS a candidate target for anticancer therapy (Camassei et al., 2003). FAS subunit alpha includes two parts, reductase and synthase, whose EC number are 1 and 2, respectively. To assess our model's ability in predicting the whole EC number sets of certain multi-functional enzyme sequences, we excluded the FAS sequences from the training data and fed those sequences to our model during testing. The outputs of our network show that mlDEEPre can exactly predict FAS's main classes which are Oxidorreductase and Transferase, being consistent with the experimental results. Furthermore, the integration of mlDEEPre and DEEPre can annotate the two sets of FAS's EC numbers correctly.

## 4. Discussion

In this paper, based on multi-label deep learning, we propose a novel method, mlDEEPre, to annotate the functionality of multi-functional enzymes. It works seemlessly with DEEPre, which enables DEEPre to perform mono-functional enzyme and multi-functional enzyme function predictions at the same time automatically. Despite of the state-of-the-art performance of mlDEEPre, this tool can still be improved in the following ways. Firstly, when designing the tool, we assume that the multiple functions of an enzyme diverge in the main class. Although that assumption holds under most circumstances, it is inevitable that there are some enzymes with different sub-class or even subsub-class functions. Those kind of enzymes need to be investigated in the future. Secondly, we also assume that the EC system remains static. Although the EC system is stable most of the time, it is a dynamic system if we exam it over a long time period, and the number of classes can increase as we discover more enzymes, which may invalidate the previous classifier. In machine learning, this problem is called class incremental learning (Li et al., 2018b). In the future, efforts will be made to enable the system to perform enzyme function prediction in dynamic labeling space. Finally, since much of the performance gain of mlDEEPre is contributed to the superior performance of deep learning in handling classification problems, some recent works in investigating the nature of deep learning (Soudry et al., 2017; Li et al., 2018c) can be helpful for further improving the performance of deep learning and thus the performance of mlDEEPre. We believe that the idea of mlDEEPre, combining multi-label learning with deep learning, can be helpful for solving other similar bioinformatics problems. For example, it has the potential to be applied to predict the properties of antibiotic-resistant genes (ARG) in multidrug-resistant pathogens (Zhu et al., 2013; Cao et al., 2018), perform classification of multicomponent transporter system (Saier et al., 2015), and predict CRISPR-Cas9 gene editing off-target regions (Fu et al., 2013; Pattanayak et al., 2013; Lin and Wong, 2018; Zhang et al., 2018).

## Data Availability Statement

The datasets for this study can be found in the http://www.cbrc.kaust.edu.sa/DEEPre/dataset.html and http://server.malab.cn/MEC/download.jsp.

## Author Contributions

YL and XG initialized and designed the project. ZZ and ST implemented the idea and run the experiments. YL and ZZ wrote the manuscript. XG and ST helped revise the manuscript. All authors provided critical feedback and helped shape the research, analysis and manuscript.

### Conflict of Interest Statement

The authors declare that the research was conducted in the absence of any commercial or financial relationships that could be construed as a potential conflict of interest.
