# Supplementary material for: mlDEEPre: Multi-Functional Enzyme Function Prediction With Hierarchical Multi-Label Deep Learning
Source: Front Genet. 2019 Jan 22;9:714. doi: 10.3389/fgene.2018.00714 (PMC6349967; doi:10.3389/fgene.2018.00714)
Supplement: Supplementary file 1 [file Image_1.pdf]

Supplementary Material:
mlDEEPre: Multi-functional enzyme function prediction with hierarchical multi-label deep learning

1 ILLUSTRATION OF KNN BASED METHODS

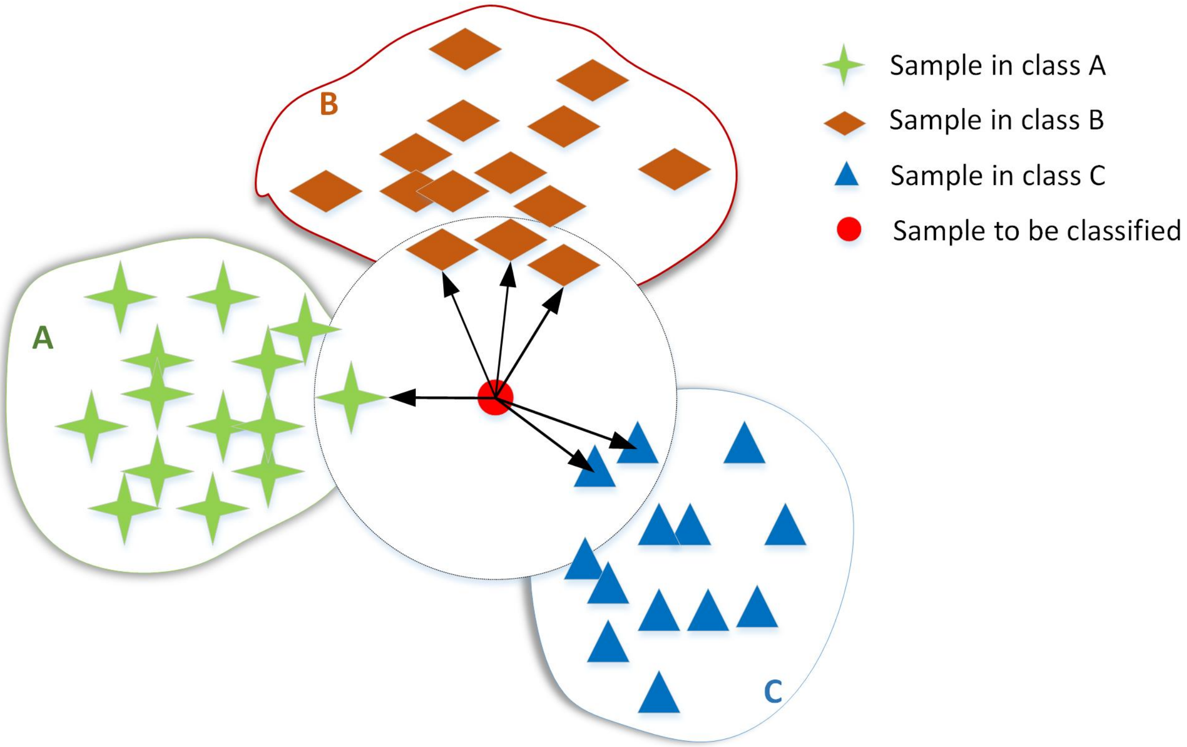

Figure S1. Illustration of KNN based methods.
